# Social Support and Subclinical Coronary Artery Disease in Middle-Aged Men and Women: Findings from the Pilot of Swedish CArdioPulmonary bioImage Study

**DOI:** 10.3390/ijerph17030778

**Published:** 2020-01-27

**Authors:** Demir Djekic, Erika Fagman, Oskar Angerås, George Lappas, Kjell Torén, Göran Bergström, Annika Rosengren

**Affiliations:** 1 Department of Cardiology, School of Medical Sciences, Örebro University, Örebro University Hospital, 701 85 Örebro, Sweden; 2 Department of Radiology, Institute of Clinical Sciences, The Sahlgrenska Academy at University of Gothenburg, Sahlgrenska University Hospital, 413 90 Gothenburg, Sweden; erika.fagman@vgregion.se; 3 Department of Molecular and Clinical Medicine, The Sahlgrenska Academy at University of Gothenburg, Sahlgrenska University Hospital, 416 85 Gothenburg, Sweden; oskar.angeras@vgregion.se (O.A.); georgios.lappas@gu.se (G.L.); goran.bergstrom@hjl.gu.se (G.B.); Annika.Rosengren@wlab.gu.se (A.R.); 4 Section of Occupational and Environmental Medicine, Sahlgrenska Academy, University of Gothenburg, 413 90 Gothenburg, Sweden; kjell.toren@amm.gu.se

**Keywords:** Social support, women, coronary artery calcium, coronary artery calcification, subclinical coronary artery disease, inflammation

## Abstract

Social support has been associated with coronary artery disease (CAD), particularly in individuals who have sustained a cardiovascular event. This study investigated the relationship between social support and subclinical CAD among 1067 healthy middle-aged men and women. Social support was assessed with validated social integration and emotional attachment measures. Subclinical CAD was assessed as a coronary artery calcium score (CACS) using computed tomography. There was no association between social support and CACS in men. In women, low social support was strongly linked to cardiovascular risk factors, high levels of inflammatory markers, and CACS > 0. In a logistic regression model, after adjustment for 12 cardiovascular risk factors, the odds ratio (95% confidence intervals) for CACS > 0 in women with the lowest social integration, emotional attachment, and social support groups (reference: highest corresponding group) were 2.47 (1.23–5.12), 1.87 (0.93–3.59), and 4.28 (1.52–12.28), respectively. Using a machine learning approach (random forest), social integration was the fourth (out of 12) most important risk factor for CACS > 0 in women. Women with lower compared to higher or moderate social integration levels were about 14 years older in “vascular age”. This study showed an association between lack of social support and subclinical CAD in middle-aged women, but not in men. Lack of social support may affect the atherosclerotic process and identify individuals vulnerable to CAD events.

## 1. Introduction

Lack of social support has been associated with higher risk for incident coronary artery disease (CAD) and higher mortality risk following a cardiac event [1]. The exact pathophysiological mechanisms whereby a lack of social support increases the risk for CAD or high social support acts as a protective factor remain unclear; however, influences on the immune, neuroendocrine, coagulation, and cardiovascular systems have been suggested [2,3,4,5,6,7,8]. Moreover, individuals who lack social support have a higher prevalence of traditional cardiovascular risk factors [6].

Coronary artery calcification (CAC) refers to the total amount of calcium deposited in the coronary arteries, with a higher coronary artery calcium score (CACS) related to increased CAD severity [9]. Previous studies investigating the relationship between CAC and social support showed heterogeneous findings, possibly because of differences in the assessment of social support, study population, and statistical analyses [10,11,12,13,14]. A better understanding of the mechanisms by which improved functioning of social networks may slow the development of CAD would advance our knowledge in this field. 

Most previous studies have focused on individuals with manifest cardiovascular disease (CVD) or have investigated the impact of social support on CVD in men only [1,2,7,8,15,16,17]. There is a lack of studies investigating the association between social support and subclinical CAD in men and women without a previous history of CAD, while considering several other psychosocial and cardiovascular risk factors. In this study, using CAC as a marker of subclinical CAD, which has been shown to correlate with the total plaque burden [9], and questionnaires previously validated and associated with CAD in Sweden [2,7,8,15,16,17,18], we used data from the pilot phase of the Swedish CArdioPulmonary bioImage Study (SCAPIS) conducted in 2012 to examine the association of social support with subclinical CAD in randomly selected healthy middle-aged men and women [19].

## 2. Methods

### 2.1. Study Population and Recruitment

**Figure 1:**
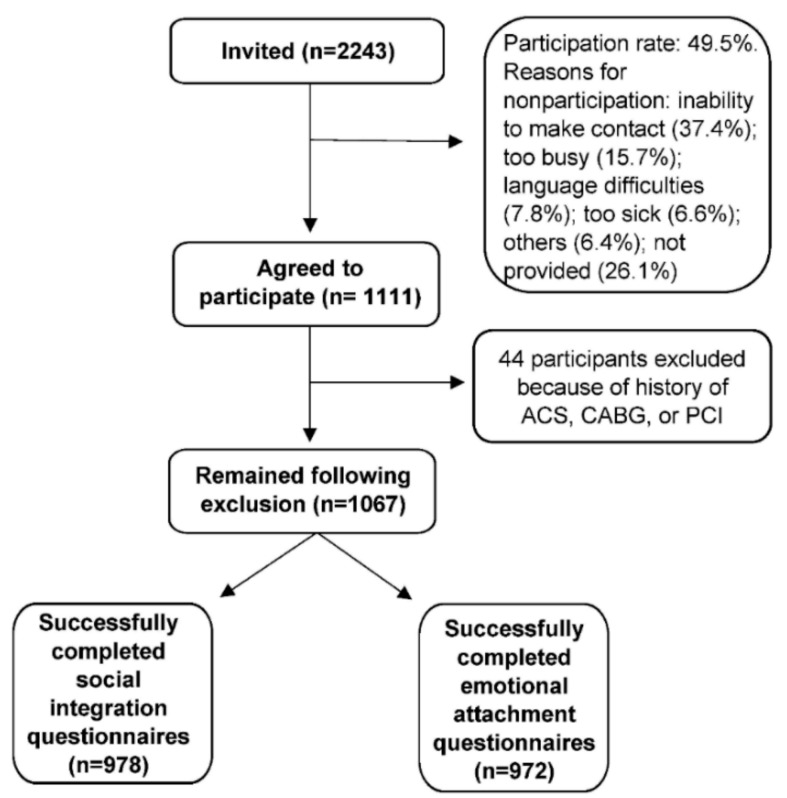
Flow chart of the study design for the pilot phase of SCAPIS and participants included in the statistical analysis. ACS, acute coronary syndrome; CABG, coronary artery bypass grafting; PCI, percutaneous coronary intervention; SCAPIS, Swedish CArdioPulmonary bioImage Study. A limited number of participants (n = 6) were excluded upon assessing social support due to missing data on emotional attachment.

Details of the study design have been published elsewhere [19]. We analyzed cross-sectional data from the pilot SCAPIS study performed at Sahlgrenska University Hospital between February and November 2012. The pilot study aimed to investigate the feasibility of SCAPIS and estimate the consequences of pathological findings identified during the investigations for the healthcare system. The participants were randomly recruited based on Swedish national identification number and official census records from residential districts of three high and low socioeconomic areas in the city of Gothenburg. A random sample of 2243 men and women were invited to participate in the pilot, and the overall participation rate was 49.5%. A flow chart of the study process is presented in Figure 1. All participants signed a detailed informed consent form before the examination at their first visit. The study was approved by the Ethics Committee of the University of Umeå (Dnr 2010-228-31M).

### 2.2. Social Support

We used questionnaires to assess social support, including quantitative, structural, qualitative, and functional aspects. Structure refers to the number of persons in the respondents’ social network and the frequency of contacts with them. Function refers to what these persons provide in terms of support. A condensed version (Table S1) of the Interview Schedule for Social Interaction, a psychiatric interview questionnaire, was used as a measure of social support [18]. This version has been previously validated and shown to be reliable [18], and it has predicted cardiovascular outcomes across multiple studies [2,7,8,15,16,17]. The instrument has two subscales: social integration and emotional attachment. Social integration describes the availability of peripheral contacts through quantitative characteristics of the network and its function, including sense of belonging, practical help, and support. Emotional attachment describes the availability of close emotional support from family and friends. There were six social integration items and six emotional attachment items (Table S1). 

### 2.3. CAC Scoring

A cardiac computed tomography (CT) scan was used for calcium scoring. The protocol has been described elsewhere [20]. In brief, the scans were performed using a standard electrocardiogram-gated protocol, and the total CACS in the coronary circulation was quantified with the Agatston method [21]. Using a replicated measure of CACS from randomly selected participants (n = 50), the kappa measure of agreement was 0.91 and 1.00 for identifying participants with a CACS of >0 and ≥ 100, respectively. Participants were classified into groups based on CACS according to recommendations developed by American College of Cardiology Foundation: no CAC (CACS = 0), mild CAC (CACS = 1–99), moderate CAC (CACS = 100–399), and severe CAC (CACS ≥ 400) [22]. This cut-off values were based on the relationship of CACS and CAD outcomes in observational studies [23]. 

CACS ≥ 100 was compared with no coronary calcification (CACS = 0), because this cut-off point is considered clinically relevant. However, individuals with mild CAC (CACS = 1–99) are at about 3.5 times higher risk of CAD events compared to individuals CACS = 0; hence, a cut-off value of CACS > 0 was also used [23,24].

### 2.4. Measurement of Standard Risk Factors

Individual socioeconomic status was assessed using data on educational level and categorized as: (1) no basic education/primary school only, (2) completed secondary school, and (3) university or college degree. Having a first-degree relative with an early cardiovascular event (mother or father with myocardial infarction or stroke before age 65 and 55 years, respectively) was defined as family history of premature CVD. Systolic and diastolic blood pressure was measured twice for each arm with an automatic device (Omron M10-IT; Omron Health Care Co., Kyoto, Japan), the mean of the two measurements was calculated, and the highest mean blood pressure was used in the analyses. Diabetes mellitus was defined as a previous diagnosis and was self-reported. Hba1c was measured in mmol/mol, and triglycerides (TG), total cholesterol, low-density lipoprotein-cholesterol (LDL-C), and high-density lipoprotein-cholesterol (HDL-C) serum levels in mmol/L, all according to standardized methods. Participants’ body weight, height, and waist circumference were measured in light clothing without shoes. White blood cell (WBC) count and high-sensitivity C-reactive protein (hs-CRP) were measured according to standard methods (×10^9^/L and in mg/L, respectively). 

Smoking status was classified as (1) never smoked, (2) active smoker (at least daily consumption of cigarettes), and (3) former/occasional smoker. Smoking burden was reported as the number of packs of cigarettes smoked per day multiplied by the number of years the participants had been a smoker (pack-year). Exercise activity was classified according to the frequency of workouts in sportswear during the last 3 months: (1) none, (2) not regularly, (3) 1/week, (4) 2–3/week, and (5) >3/week. Current depression was defined if participants reported at least five of the following criteria (including the first criterion) during the last year for a period of 2 weeks: (1) decreased mood or feeling of sadness or emptiness, (2) decreased interest or pleasure in most of the activities, (3) weight or appetite change, (4) insomnia, (5) fatigue or loss of energy, (6) feelings of guilt or worthlessness, (7) concentration problems, and (8) thoughts of death. Marital status was categorized as: (1) not married and living alone, (2) married or cohabiting, (3) divorced, and (4) widow/widower.

### 2.5. Statistical Methods

We applied both traditional statistical methods and machine learning to assess the strength of association and the relative importance between CAC and CVD risk factors. A multiple logistic regression model was fitted to the data to assess the statistical association between the outcome CAC and social support independent of age, sex, and cardiovascular and psychosocial factors. Model estimated odds ratios (ORs) and 95% confidence intervals (CIs) are shown. We compared the relative importance of each predictor for CAC in the logistic regression model. The goodness of the fit was determined with the McFadden pseudo-R2. R2 indicates how much of the variation in the dependent variable (i.e., CACS > 0) is explained by the independent variables (i.e., CVD risk factors) of a regression model and ranges from 0 to 1. The McFadden pseudo-R2 values between 0.2 and 0.4 is considered to be an excellent fit of the logistic regression model and is equivalent to a value of between 0.7 and 0.9 in a linear regression model [25]. The decision to enter the outcome as a binary outcome, comparing CACS = 0 and CACS>0 or CACS = 0 and CACS≥ 100, in the analysis instead of as a continuous outcome was because of the extreme skewness of the continuous CACS variable. The statistical model was fitted separately by sex to account for the potential difference in the burden of cardiovascular risk factors between men and women. Social integration variables were summed to obtain an overall score, with the scores appeared to be normally distributed on a histogram (Figures S1 and S2). However, the Shapiro–Wilkes p-value was < 0.05, suggesting that the scores was not normally distributed. The scores were divided into three groups: 25% in the lower group (lower quartile), 50% in the middle group (intermediate two quartiles), and 25% in the highest group (upper quartile). The highest quartile of social integration was used as the reference. Emotional attachment variables were also summed, but these were highly skewed toward the left (the highest values) on the histogram (Figures S3 and S4). Emotional attachment was categorized into three groups: those with scores of 0–4 in the lowest group, scores of 5 in the middle group, and scores of 6 in the highest group. These cut-off values have been used in previous observational studies and were associated with higher risk for CAD atherosclerotic burden and CAD events [2,7,8,15,16,17]. For social integration or for emotional attachment, a higher score was equivalent to a higher degree of social support; however, the assumption of truly interval scales is not fully satisfied, as the meaning of a change in one score is not the same at all points of the scale. Thus, the analysis of the grouped categories of social support as ordered scales is more statistically correct. Participants with low levels of social integration (score 1–10) and emotional attachment (score 1–4) were also combined to evaluate whether a combination of the two subscales was more strongly associated with CAC. This group is referred to as low social support throughout the text. Participants with missing data on emotional attachment (n = 6) were excluded in the statistical analysis upon assessing social support. Model 1 was adjusted for age and Model 2 was further adjusted for CVD risk factors: family history of premature CVD, diabetes, systolic and diastolic blood pressure, HbA1C, LDL-C, HDL-C, TG, obesity, and total smoking burden (Table 3). Additionally, we adjusted for psychosocial factors in Model 3.

Random forest is a robust and nonparametric machine learning statistical method that optimizes predictive accuracy by fitting an ensemble of trees, and it was used to stabilize model estimates [26]. Random forest uses all available variables in a model to ascertain which variables contribute mostly to the prediction. We used two separate approaches to explore the random forest selection process: variable importance and minimal depth. In variable importance, predictors are determined by testing the forest prediction under alternative data settings, ranking the most important risk factors according to their impact on predictive ability of the forest [26]. Minimal depth measures the important risk factors by averaging the depth of the first split for each variable over all the trees within the forest [27]. The data was divided into training and testing datasets compromising 70% and 30% of the total sample size, respectively. Then, the test dataset was used to determine the performance of the random forest to predicted CACS > 0, using all predictors in the model. Model 4 shows the accuracy of the random forest to prediction CACS > 0, when social integration, emotional attachment, or social support was added to a CVD risk factor model (Table 3). Finally, to aid interpretation of the random forest results, partial dependence plots were produced to show the interaction effects among the most important cardiovascular risk factors. All analyses were performed using R software, version 3 (moonBook, ggplot, caret, randomForest, randomForestSRC and plotmo packages; R Foundation for Statistical Computing, Vienna, Austria). 

## 3. Results

### 3.1. Study Cohort

The final sample from the pilot study included 1111 men and women aged 50–64 years, recruited from three high and three low socioeconomic residential areas in the city of Gothenburg (570,000 inhabitants). After excluding 44 participants with a history of CVD, 978 and 972 of the remaining 1067 participants completed the social integration and emotional attachment questionnaires, respectively (Figure 1). 

### 3.2. Social Support and Clinical Risk Factors

Table 1 shows cardiovascular and psychosocial characteristics by sex and social integration. In men, lower social integration was associated with diabetes mellitus, active smoking, and lower physical activity. Women with lower social integration were more likely to have a family history of premature CVD, higher mean body mass index (BMI), and waist circumference; have diabetes mellitus; be active smokers; and be less physically active. Laboratory analyses in women showed higher median WBC count, higher median hs-CRP levels, higher mean HbA_1C_, higher median TG levels, and lower median HDL-C levels in those with low levels of social integration.

Table 2 displays participants’ cardiovascular and psychosocial characteristics according to different levels of emotional attachment. Men with low, compared to high, emotional attachment were more often smokers, but no other CVD risk factor or biochemical marker differed significantly by level of emotional attachment. Women with low, compared to high, emotional attachment more often had a family history of premature CVD, higher mean BMI, larger waist circumference, higher smoking burden, and lower exercise activity. Biochemical analyses showed higher median WBC count and hs-CRP levels, higher mean HbA1C, lower median HDL-C and higher median TG levels with lower emotional attachment. 

There were strong intercorrelations between social integration and emotional attachment scales (Table S2).

### 3.3. Social Support and Prevalence of CAC

**Figure 2:**
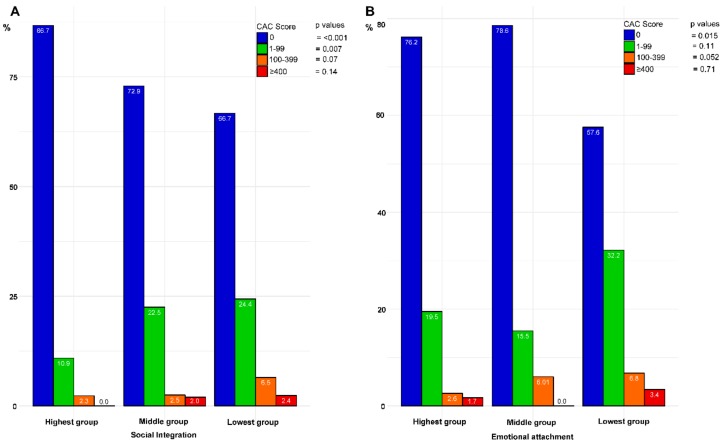
Bar plots of the prevalence of coronary artery calcium score (CACS) = 0, CACS = 1–99, CACS = 100–399, and CACS ≥ 400 according to different levels of social integration (**A**) and emotional attachment (**B**) in women. Data presented as %. P-values calculated by chi-squared tests for trend.

In men, neither social integration nor emotional attachment was significantly associated with a higher prevalence of CAC scores (Figure S5). Among women, the prevalence of no CAC was 86.7%, 72.9%, and 66.7% in the highest, middle, and lowest levels of social integration, respectively (Figure 2A). The lowest emotional attachment group also had a lower prevalence of no CAC (57.6%) compared with the highest group (76.2%) (Figure 2B).

Considering social support, the combined scale of social integration and emotional attachment, the proportion of women with CACS ≥ 400 was significantly higher in women with lower (6.5%), compared to higher (0.0%), levels of social support (Figure S6A), whereas the proportion with no CAC was 86.1% in the highest group, compared to 51.6% in the lowest group (*p* for trend < 0.001). No significant differences were seen for men (Figure S6B)

### 3.4. Association of Social Support and CAC

In the age and sex-adjusted logistic regression model for the total study population, combining men and women, the odds ratio (OR) for CACS > 0 comparing the highest and the lowest social integration group was 1.56, 95% CI: 1.04–2.34; *p* = 0.031 (Table 3). However, there was no significant association when the lowest and highest social integration or emotional attachment groups were compared after adjustment for CVD risk factors (Table 3).

In men, neither CACS > 0 nor CACS ≥ 100 was associated with social integration or emotional attachment after adjusting for age (Table 3, Table S3) in the logistic regression model. However, the pseudo R2, as an expression of goodness of fit and how much of the variation in CACS was explained by the covariates in the model, increased from 0.14 to 0.20 and 0.24 when social integration and social support variables were added to CVD risk factor model, respectively.

Random forest, a machine learning statistical method, was used to optimize the predictive accuracy of CACS > 0 and to select the CVD risk factors that contribute mostly to the prediction of CACS > 0. The accuracy of the random forest in predicting CACS > 0 was 0.52 (95% CI: 0.42–0.62: *p* = 0.27) in the social integration and CVD risk factor model in men (Table 3).

**Figure 3:**
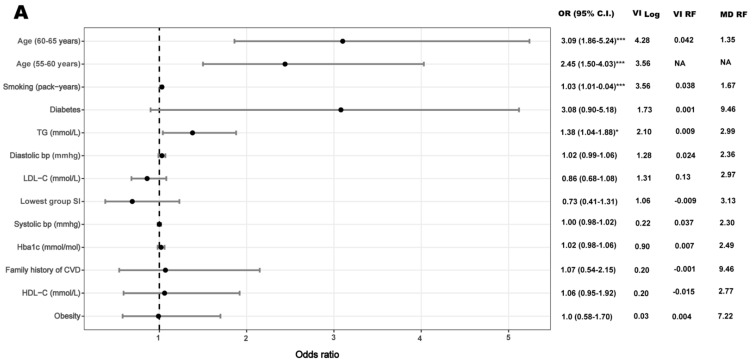

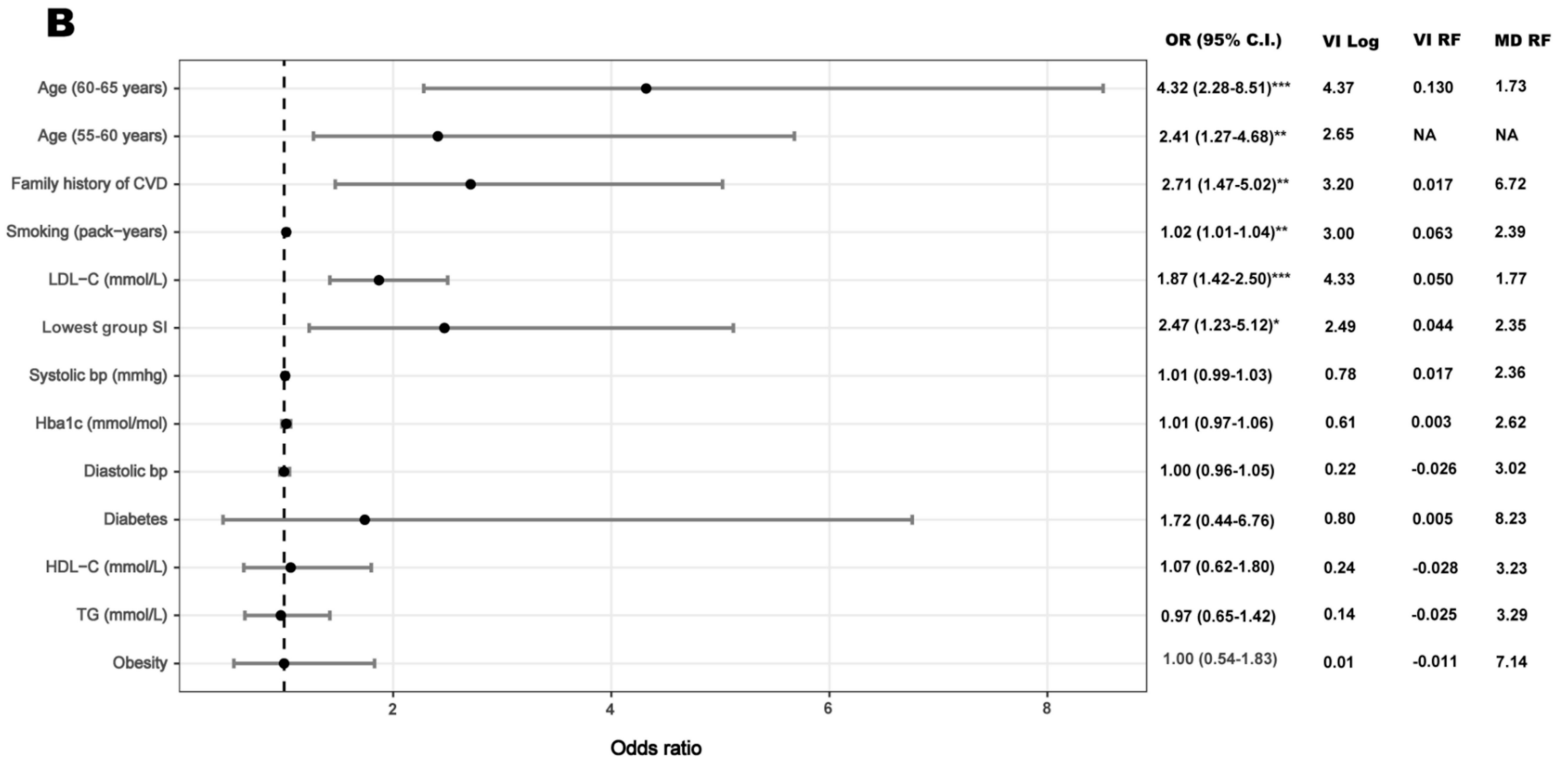
Relationship of cardiovascular risk factors and social integration with coronary artery calcium score >0 obtained from the logistic regression models and machine learning analysis. Variable importance (VI RF) and minimal depth (MD RF) were used to explore the relative importance of cardiovascular risk factors in the prediction of coronary artery calcium by random forest, from the most important (highest values) to the least important (lowest values) for VI RF and vice versa for MD RF. Correspondingly, we determined the relative importance of predictors in the logistic regression models (VI Log); from most important (highest values) to the least important (lowest values) predictors of CACS>0. Results are displayed in men (**A**) and women (**B**), separately. Bp: blood pressure; C.I.: confidence interval; CVD: cardiovascular disease; HDL-C: high-density lipoprotein cholesterol; LDL-C: low-density lipoprotein cholesterol; OR: odds ratio; SI: social integration; TG: triglycerides. * *p* < 0.05, ** *p* < 0.01,*** *p* < 0.001. NA; Not applicable, age was used as a continuous covariate in the machine learning analysis.

In women, the OR for CACS > 0 was 3.21 (95% CI: 1.71–6.26; *p* ≤ 0.001) in the lowest social integration group and 2.21 (95% CI: 1.24–4.12; *p* = 0.009) in the middle group compared with the highest group after adjusting for age (Table 3). With further adjustment for CVD risk factors, the OR of CACS > 0 was 2.47 (95% CI: 1.23–5.12; *p* = 0.013) in the lowest and 1.82 (95% CI: 0.97–3.55; *p* = 0.067) in the middle social integration groups compared with the highest group (Table 3, Figure 3B). This association remained significant after further adjustment for marital status, depression, education, and residential area SES (Table 3). The pseudo R2 increased from 0.18 to 0.26 when social integration was added to the CVD risk factor model. 

**Figure 4:**
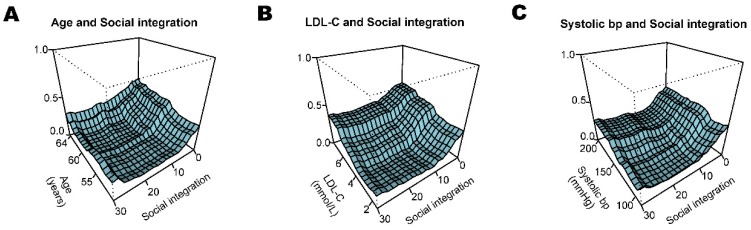
Three-dimensional partial dependence plots generated from the random forest models in women. We visualize the most important interactions between two cardiovascular risk factors (X_1_ and X_2_ axes) and their impact on coronary artery calcium score >0 (Y-axis) while adjusting for 12 cardiovascular risk factors in the random forest models. Interaction effects between social integration and (**A**) age, (**B**) LDL-C, and (**C**) systolic bp. Bp: blood pressure; LDL-C: low-density lipoprotein cholesterol. Y-axis denotes probability of coronary artery calcium score >0.

In women, the accuracy of the random forest in predicting CACS > 0 was 0.80 (95% CI: 0.71–0.87; *p* = 0.006) in the social integration and CVD risk factors model (Table 3). Thus, 80% of the participants in the tested dataset were correctly predicted. We applied two separate approaches, variable importance and minimal depth, to evaluate which covariates contributed to the predictive accuracy of CACS. Out of 12 cardiovascular risk factors, low levels of social integration was the fourth most important predictor of CACS > 0 according to the random forest model in women using both approaches (Figure 3B). To visualize the complex interaction effects of social integration (fourth most important predictor of the random forest) with the most important CVD risk factors, we constructed three-dimensional partial dependence plots (Figure 4). We found that there were interaction effects between social integration and age, LDL-C, and systolic blood pressure on CACS > 0 in women. Age increased the probability of CACS > 0 in middle-aged women with lower levels of social integration, but not for women with moderate or high levels social integration (Figure 4A). Fifty-year-old women with lower social integration levels had approximately the same prevalence of CACS > 0 as 64-year-old women with higher or moderate social integration levels.

Women with lower compared to high or moderate social integration levels had a high probability of CACS > 0, despite LDL-C levels (<3 mmol/L) or systolic blood pressure (100–130 mmHg) within the normal range (Figure 4B and C).

CACS ≥ 100 in women was associated with low social integration (OR = 4.94, 95% CI: 1.43–22.90; *p* = 0.02) for the lowest group compared with the highest group; however, this relationship was no longer significant after further adjustment for CVD risk factors (Table S3).

In women, the OR for CACS > 0 was 2.46 (95% CI: 1.35–4.46; *p* = 0.003) in the lowest emotional attachment group compared with the highest group, after adjusting for age (Table 3). With further adjustment for CVD risk factors, the OR for CACS > 0 was 1.83 (95% CI: 0.93–3.59; *p* = 0.07). In women, the addition of emotional attachment to the CVD risk factor model led to an increase in the pseudo R2, from 0.18 to 0.24.

In women, with adjustment for CVD risk factors and psychosocial factors, the OR was 4.28 (1.52–12.28; *p* = 0.006) and 4.20 (1.31–13.60; *p* = 0.015), respectively, for the lowest compared with the highest social support groups. The pseudo R2 increased from 0.18 to 0.30 when social support was added to the CVD risk factor model in women.

## 4. Discussion

In this cross-sectional study involving middle-aged men and women without a previous history of CAD, we investigated the relationship between social support and subclinical CAD. Our results suggest that lack of social support may be associated with subclinical CAD in women, but not in men. This relationship in women was not explained by higher levels of CVD risk factors. The pseudo R2, as an expression of goodness of fit and how much of the variation in subclinical CAD is explained by the covariates in the logistic regression model, increased from 0.18 to 0.26 when social integration variables were added to the CVD risk factor model. The importance of social integration as a predictor of subclinical CAD was also demonstrated with a machine learning approach. The accuracy of the random forest in predicting subclinical CAD in women was high and improved when social integration was added to the model. In women, social integration was the fourth most important factor of subclinical CAD out of 12 CVD risk factors. Interestingly, social integration interacted with traditional cardiovascular risk factors in women, and we found an increased probability of subclinical CAD in women who scored below 10 on the social integration scale. Thus, both traditional and machine learning approaches suggests that social integration affects subclinical CAD independent of CVD risk factor.

Social support was measured by social integration and emotional attachment. These subscales describe perceived access to peripheral contacts through quantitative characteristics of networks and their function. We were able to adjust for a broad range of CVD risk factors and, in addition, we adjusted for other psychological and social factors such as depression, marital status, education, and residential area SES. After adjusting for major CVD risk factors and these factors, the risk for subclinical CAD remained significantly higher in women in the lowest social integration group compared with the highest group. Women in the lowest emotional attachment group also had a higher risk for subclinical CAD, although this relationship was not significant after adjusting for CVD risk factors. However, the CIs were wide, and there was a corresponding lack of power. 

In men, we did not demonstrate any association between the social support measures and either CACS >0 or a CACS≥100. There may be a number of reasons for this discrepancy; first, fewer men than women successfully completed questionnaires for social support, and a higher proportion of those with manifest CAD were men. Second, social support may be a more important CVD risk factor among middle-aged women compared with men of the same age, as men tend to develop traditional CVD risk factors and CVD earlier in life. Finally, women may be better able to express their emotions and/or respond to questionnaires more honestly compared with men [28]. 

Previous prospective reports indicated that a lack of social support, assessed by the same scale as in our study, was predictive of CAD events in middle-aged men independent of CVD risk factors [2,8,15]. A lack of social support has also been related to the severity of CAD assessed by coronary angiography in young and middle-aged women hospitalized for a suspected CAD event [16]. In addition, during follow up, women with lower social support had a worse prognosis and recurrent CAD events [17]. Few previous studies have investigated the relationship between social support and subclinical CAD (measured as CACS), and findings have been heterogeneous. In the Coronary Artery Risk Development in Young Adults study, low social cohesion was independently associated with CAC in young and middle-aged women but not in men [10]. Kop et al. studied the relationship between social networks and CAC; however, social networks were mainly defined by marital status, and being single or widowed was independently associated with CAC in both men and women [11]. Another study investigated 528 asymptomatic men and women aged 53–76 years, and no association between social support and CAC was observed [12]. In a longitudinal study, no significant relationship was found between social support and CAC progression [13].

Although the mechanisms by which lack of perceived social support leads to the development of CAD remain poorly understood, some pathways have been suggested (biological, psychosocial, physiological) [3,4,5,6,7,8,29,30]. Having an extended network may provide information regarding visiting a physician or lifestyle recommendations that positively influence health. Previous studies have linked social isolation with adverse behaviors such as smoking, excessive alcohol consumption, lower physical activity, and an unhealthy diet [6]. This is supported by our study, in which women in the lowest social support group displayed these adverse behaviors. However, in our study, the impact of social support on CVD risk factors did not fully account for this relationship, as adjusting for CVD risk factors did not significantly attenuate the OR for subclinical CAD. 

Other reports have found explanations related to physiological mechanisms, such as neuroendocrine and immunological dysfunction. For example, some studies have suggested that a lack of social networks might alter the hypothalamus–pituitary–adrenal axis, resulting in WBC glucocorticoid receptor resistance and leading to the enhanced formation of pro-inflammatory cytokines [3]. In previous studies, socially integrated individuals had lower plasma levels of interleukin (IL)-6 (a cytokine involved in the production of CRP in liver) and CRP. Even though some existing studies have reported a relationship between social isolation and elevated CRP in middle-aged persons, others only found elevated plasma CRP levels in older socially isolated men but not younger men and women [26,27]. Interestingly, in our study, women in the lowest social support group had significantly higher levels of hs-CRP and a higher WBC count. These observations may be consistent with our finding that women in the lowest social support group had higher levels of low-grade systemic inflammation, which together with an increased burden of CVD risk factors may contribute to the development of subclinical CAD.

Nevertheless, the presence of subclinical CAD, measured as coronary calcium, can be used to determine the “vascular age” of an individual [31]. For instance, smoking women are about 10 years older in “vascular age” compared to non-smoking women [32]. Similarly, women with diabetes, compared to non-diabetics are about 13 years older in “vascular age” [32]. The current study adds to the literature that women with lower compared to higher or moderate social integration levels are about 14 years older in “vascular” age. Hence, low social integration may accelerate vascular aging in women.

The present study has several strengths, including a representative sample from the population of middle-aged men and women, the used of validated questionnaires to assess social support, and the inclusion of a large number of cardiovascular and psychosocial covariates in the adjusted models. Furthermore, we used two statistical approaches, traditional and machine learning methods, to assess the association of social support with subclinical CAD. This study also has two main limitations that need to be discussed. Firstly, the cross-sectional study design and all information being gathered at one specific point in time make assumptions about causality not possible. Such assumptions would require that lower levels of social support were found to precede CAC, using a longitudinal design. Secondly, caution must be applied when extrapolating the present findings to men and women outside the ages of the study subjects and to other populations outside Sweden.

## 5. Conclusions

In this study, we found an association between lack of social support and subclinical CAD in middle-aged women but not in men. This association was independent of CVD risk factors and may indicate an effect on the atherosclerotic process accelerating vascular aging. Lack of social support may help to identify individuals vulnerable to CAD events, and these subjects should be screened for traditional CVD risk factors. 

## Figures and Tables

**Table 1:** Characteristics of cardiovascular risk factors and psychosocial factors according to sex and social integration.

	Men				Women			
Social Integration	Highest (n = 129)	Middle (n = 249)	Lowest (n = 105)	*p*	Highest (n = 128)	Middle (n = 244)	Lowest (n = 123)	*p*
Age, years, mean ± SD	57.4 ± 4.2	57.7 ± 4.5	57.8 ± 4.7	0.56	56.9 ± 4.0	58.0 ± 4.4	57.3 ± 4.2	0.42
Family history of premature CVD ^1)^, n (%)	15 (11.6)	24 (9.6)	7 (6.7)	0.20	5 (3.9)	36 (14.8)	24 (19.5)	<0.001
Diabetes mellitus ^†^, n (%)	3 (2.3)	20 (8.0)	12 (11.4)	0.007	1 (0.8)	12 (4.9)	9 (7.3)	0.01
Systolic BP, mmHg ^†^, mean ± SD	129 ± 16.3	131 ± 17.8	130 ± 16.1	0.88	123 ± 16.5	126 ± 18.2	123 ± 16.8	0.92
Diastolic BP, mmHg ^†^, mean ± SD	78 ± 9.1	79 ± 9.1	79 ± 8.8	0.78	73 ± 9.0	74 ± 9.2	72 ± 8.2	0.69
BMI, kg/m^2 †^, mean ± SD	27.4 ± 3.0	27.9 ± 4.5	27.7 ± 4.0	0.54	25.6 ± 4.0	27.1 ± 5.2	27.3 ± 5.1	0.011
Waist circumference, cm ^†^, mean ± SD	99.9 ± 8.8	100.5 ± 11.8	100.6 ± 10.9	0.64	86.8 ± 11.8	90.8 ± 12.8	92.0 ± 12.9	0.002
Active smoker^¤^, n (%)	15 (11.6)	35 (14.1)	23 (21.9)	0.033	11 (8.6)	30 (12.3)	30 (24.4)	<0.001
Smoking burden^¤^, pack-years, median [IQR]	0 [0;15.8]	5 [0;18.8]	5 [0;20.0]	0.13	0 [0;4.2]	1.43 [0;15.0]	5.0 [0;20.0]	<0.001
No exercise activity ^‡^, n (%)	35 (27.1)	88 (35.3)	47 (44.8)	0.005	27 (21.1)	63 (25.8)	67 (54.5)	<0.001
Low SES residential area, n (%)	37 (28.7)	125 (50.2)	71 (67.6)	<0.001	38 (29.7)	110 (45.1)	83 (67.5)	<0.001
University degree ^†^, n (%)	55 (42.6)	83 (33.3)	25 (23.8)	0.002	84 (65.6)	95 (38.9)	29 (23.6)	<0.001
Depression ^†^, n (%)	2 (1.6)	15 (6.0)	15 (14.3)	<0.001	7 (5.5)	29 (11.9)	30 (24.4)	<0.001
Married/ Cohabiting ^†^, n (%)	117 (90.7)	197 (79.1)	67 (63.8)	<0.001	103 (80.5)	186 (76.2)	74 (60.2)	<0.001
WBC count, ×10^9^/L ^†^, median [IQR]	5.7 [4.8; 6.7]	5.9 [5.0; 7.0]	5.8 [4.8; 7.0]	0.003	5.4 [4.4; 6.4]	5.7 [4.3; 6.8]	6.4 [5.0; 7.5]	<0.001
hs-CRP, mg/L ^†^, median [IQR]	1.20 [0.63; 2.10]	1.20 [0.60; 2.50]	1.60 [0.72; 2.80]	0.089	0.98 [0.51; 2.10]	1.40 [0.65; 3.10]	1.70 [0.99; 3.55]	<0.001
HbA_1c,_ mmol/mol ^†^, mean ± SD	35.6 (4.66)	36.8 (7.98)	37.3 (10.0)	0.094	34.4 (3.37)	36.2 (6.54)	37.3 (9.49)	<0.001
Total cholesterol, mmol/L ^†^, mean ± SD	5.77 ± 1.07	5.49 ± 1.08	5.72 ± 1.01	0.67	5.93 ± 0.89	6.00 ± 1.00	5.82 ± 1.08	0.46
LDL-C, mmol/L ^†^, mean ± SD	3.90 ± 0.93	3.67 ± 0.98	3.88 ± 0.99	0.76	3.72 ± 0.83	3.82 ± 0.93	3.72 ± 1.00	0.94
HDL-C, mmol/L ^†^, median [IQR]	1.40 [1.20; 1.80]	1.40 [1.20; 1.70]	1.40 [1.20; 1.70]	0.32	2.00 [1.60; 2.30]	1.90 [1.55; 2.30]	1.70 [1.40; 2.20]	0.005
Triglycerides, mmol/L ^†^, median [IQR]	1.20 [0.91; 1.60]	1.20 [0.89; 1.70]	1.20 [0.96; 2.00]	0.25	0.91 [0.67; 1.30]	0.99 [0.76; 1.40]	1.10 [0.83; 1.85]	<0.001

Data are presented as n (%), mean ± standard deviation (SD), or median [interquartile range]. CVD, cardiovascular disease; BP, blood pressure; LDL-C, low-density lipoprotein cholesterol; HDL-C, high-density lipoprotein cholesterol; BMI, body mass index; WBC, white blood cell; hs-CRP, high sensitivity C-reactive protein. ^†^ Missing values: minor discrepancies explained by a limited number of missing participants (<10); **^1)^** 24 missing; ^‡^ 15 missing. Social integration was categorized into quartiles with scores of 0–10 (lowest group), 11–19 (middle group) and 20–30 (highest group).

**Table 2:** Characteristics of cardiovascular risk factors and psychosocial factors according to sex and emotional attachment.

	Men				Women			
Emotional attachment	Highest (n = 269)	Middle (n = 108)	Lowest (n = 100)	*p*	Highest (n = 349)	Middle (n = 84)	Lowest (n = 59)	*p*
Age, years, mean ± SD	57.7 ± 4.5	57.3 ± 4.5	58.2 ± 4.3	0.66	57.6 ± 4.4	57.3 ± 3.8	57.8 ± 4.4	0.95
Family history of premature CVD ^1)^, n (%)	25 (9.3)	7 (6.5)	10 (10.0)	0.98	37 (10.6)	18 (21.4)	13 (22.0)	0.002
Diabetes mellitus ^†^, n (%)	21 (7.8)	6 (5.6)	10 (10.0)	0.65	16 (4.6)	2 (2.4)	4 (6.8)	0.76
Systolic BP, mmHg ^†^, mean ± SD	130 ± 18.2	130 ± 16.0	130 ± 16.7	0.82	125 ± 18.4	124 ± 15.6	123 ± 14.3	0.85
Diastolic BP, mmHg ^†^, mean ± SD	79 ± 9.4	78 ± 8.3	79 ± 9.1	0.73	73 ± 9.3	73 ± 8.6	73 ± 8.7	0.97
BMI, kg/m^2 †^, mean ± SD	27.6 ± 4.1	27.8 ± 3.6	27.8 ± 4.2	0.63	26.3 ± 4.4	28.3 ± 5.6	27.4 ± 5.8	0.004
Waist circumference, cm ^†^, mean ± SD	100.1 ± 10.9	100.5 ± 10.0	101.1 ± 11.3	0.57	88.9 ± 11.7	94.0 ± 14.0	92.9 ± 14.9	0.002
Active smoker ^†^, n (%)	32 (11.9)	16 (14.8)	23 (23.0)	0.010	41 (11.7)	18 (21.4)	10 (16.9)	0.070
Smoking burden, pack-year, median [IQR] ^†^	2.5 [0; 16.5]	5.18 [0; 19.3]	10.0 [0; 22.5]	0.04	0 [0; 11.5]	4.9 [0; 15.2]	4.4 [0; 23.1]	0.005
No exercise activity ^‡^, n (%)	94 (34.9)	36 (33.3)	39 (39.0)	0.56	89 (25.5)	41 (48.8)	30 (50.8)	<0.001
Low SES residential area, n (%)	126 (46.8)	50 (46.3)	57 (57.0)	0.12	156 (44.7)	44 (52.4)	35 (59.3)	0.022
University degree ^†^, n (%)	90 (33.5)	35 (32.4)	35 (35.0)	0.84	157 (45.0)	28 (33.3)	21 (35.6)	0.055
Depression ^†^, n (%)	14 (5.2)	7 (6.5)	11 (11.0)	0.058	18 (5.2)	16 (19.0)	27 (45.8)	<0.001
Married/cohabiting ^†^, n (%)	234 (87.0)	81 (75.0)	64 (64.0)	<0.001	287 (82.2)	47 (56.0)	29 (49.2)	<0.001
WBC count, ×10^9^/L ^†^, median [IQR]	5.8 [4.8; 7.0]	5.9 [5.0; 7.0]	5.7 [4.8; 6.7]	0.42	5.4 [4.4; 6.4]	5.7 [4.3; 6.8]	6.4 [5.0; 7.5]	0.008
hs-CRP, mg/L ^†^, median [IQR]	1.55 [0.68; 2.85]	1.05 [0.72; 2.35]	1.30 [0.61; 2.40]	0.41	1.20 [0.58; 2.60]	1.90 [0.90; 3.30]	1.80 [0.96; 3.60]	<0.001
HbA1c,mmol/mol ^†^, mean ± SD	36.7 (7.24)	35.9 (6.28)	38.0 (10.47)	0.26	37.0 (5.86)	36.3 (5.37)	37.7 (12.2)	0.043
Total cholesterol, mmol/L ^†^, mean ± SD	5.67 ± 1.11	5.63 ± 1.13	5.54 ± 1.04	0.21	6.15 ± 1.08	5.79 ± 0.94	5.94 ± 1.01	0.97
LDL-C, mmol/L ^†^, mean ± SD	3.84 ± 1.05	3.83 ± 1.03	3.70 ± 0.93	0.16	3.98 ± 0.95	3.70 ± 0.86	3.76 ± 0.92	0.40
HDL-C, mmol/L ^†^, median [IQR]	1.40 [1.20; 1.70]	1.40 [1.20; 1.70]	1.40 [1.20; 1.70]	0.53	1.90 [1.50; 2.30]	1.80 [1.40; 2.10]	1.70 [1.40; 2.10]	0.006
Triglycerides, mmol/L ^†^, median [IQR]	1.20 [1.00; 2.00]	1.20 [0.95; 1.70]	1.20 [0.87; 1.60]	0.096	0.98 [0.74; 1.40]	1.10 [0.80; 1.65]	1.10 [0.89; 1.50]	0.010

Data presented as n (%), mean ± standard deviation, or median [interquartile range]. CVD, cardiovascular disease; BP, blood pressure; LDL-C, low-density lipoprotein cholesterol; HDL-C, high-density lipoprotein cholesterol; BMI, body mass index; WBC, white blood cell; hs-CRP, high sensitivity C-reactive protein. *p*-values calculated using chi-square tests of trend for categorical variables and Spearman’s rank correlation tests for continuous variables. ^†^ Missing values: minor discrepancies explained by a limited number of missing participants (<10); ^1)^24 missing; ^‡^ 15 missing. Emotional attachment was categorized into three groups: those with scores of 0–4 in the lowest group, a score of 5 in the middle group, and a score of 6 in the highest group.

**Table 3:** Logistic regression and machine learning models for coronary artery calcium score >0 by social integration, emotional attachment, and social support.

	Model 1 *		Model 2 ^†^		Model 3 ^‡^		Model 4 +	
	OR (95% CI)	*p*	OR (95% CI)	*p*	OR (95% CI)	*p*	Accuracy (95% CI)	*p*
**Social integration**								
Highest group (total)	1.00		1.00		1.00			
Middle group (total)	1.46 (1.04–2.06)	0.031	1.32 (0.92–1.90)	0.13	1.29 (0.88–1.88)	0.19		
Lowest group (total)	1.56 (1.04–2.34)	0.031	1.24 (0.81–1.91)	0.32	1.23 (0.77–1.97)	0.39	0.71 (0.64–0.76)	0.07
Highest group (women)	1.00		1.00		1.00			
Middle group (women)	2.21 (1.24–4.12)	0.009	1.82 (0.97–3.55)	0.067	1.85 (0.95–3.74)	0.077		
Lowest group (women)	3.21 (1.71–6.26)	<0.001	2.47 (1.23–5.12)	0.013	2.51 (1.16–5.61)	0.022	0.80 (0.71–0.87)	0.006
Highest group (men)	1.00		1.00		1.00			
Middle group (men)	1.16 (0.74–1.80)	0.52	1.02 (0.64–1.64)	0.93	1.02 (0.64–1.67)	0.93		
Lowest group (men)	0.92 (0.54–1.57)	0.76	0.73 (0.41–1.31)	0.29	0.75 (0.39–1.43)	0.38	0.52 (0.42–0.62)	0.27
**Emotional attachment**								
Highest group (total)	1.00		1.00		1.00			
Middle group (total)	1.26 (0.90–1.76)	0.18	1.08 (0.76–1.55)	0.66	1.19 (0.82–1.73)	0.35		
Lowest group (total)	1.64 (1.15–2.35)	0.007	1.31 (0.88–1.93)	0.18	1.34 (0.88–2.05)	0.17	0.63 (0.57–0.69)	0.10
Highest group (women)	1.00		1.00		1.00			
Middle group (women)	0.92 (0.50–1.64)	0.78	0.77 (0.40–1.46)	0.44	0.89 (0.43–1.78)	0.75		
Lowest group (women)	2.46 (1.35–4.46)	0.003	1.83 (0.93–3.59)	0.07	1.73 (0.80–3.70)	0.16	0.77 (0.68–0.84)	0.005
Highest group (men)	1.00		1.00		1.00			
Middle group (men)	1.15 (0.72–1.84)	0.56	1.11 (0.68–1.83)	0.68	1.14 (0.69–1.92)	0.61		
Lowest group (men)	0.90 (0.56–1.44)	0.65	0.72 (0.43–1.21)	0.21	0.73 (0.42–1.26)	0.26	0.72 (0.62–0.79)	0.07
**Social support**								
Highest group (total)	1.00		1.00		1.00			
Middle group (total)	1.45 (1.00–2.10)	0.049	1.25 (0.85–1.86)	0.26	1.26 (0.84–1.90)	0.28		
Lowest group (total)	1.83 (1.02–3.30)	0.043	1.22 (0.64–2.31)	0.54	1.20 (0.60–2.40)	0.61	0.65 (0.58–0.71)	0.08
Highest group (women)	1.00		1.00		1.00			
Middle group (women)	2.03 (1.13–3.86)	0.023	1.64 (0.86–3.30)	0.14	1.80 (0.89–3.77)	0.11		
Lowest group (women)	5.71 (2.30–14.52)	<0.001	4.28 (1.52–12.28)	0.006	4.20 (1.31–13.60)	0.015	0.77 (0.67–0.84)	0.003
Highest group (men)	1.00		1.00		1.00			
Middle group (men)	1.14 (0.69–1.87)	0.60	0.98 (0.57–1.65)	0.94	1.01 (0.58–1.75)	0.96		
Lowest group (men)	0.86 (0.41–1.81)	0.69	0.54 (0.23–1.22)	0.14	0.61 (0.24–1.48)	0.27	0.67 (0.57–0.76)	0.09

OR, odds ratio; CI, confidence interval. OR, 95% CI, and *p*-values for coronary artery calcification for different levels of social integration and emotional attachment in the total study population and by sex after accounting for cardiovascular disease risk factors and psychosocial factors. * Model 1, adjusted for age and sex. ^†^ Model 2, adjusted for age, sex, and cardiovascular risk factor, including family history of premature cardiovascular disease, burden of smoking (pack-years), systolic blood pressure, diastolic blood pressure, diabetes, obesity, HbA_1C_, low-density lipoprotein cholesterol, high-density lipoprotein cholesterol, and triglycerides. ^‡^ Model 3, adjusted for age, sex, cardiovascular risk factors, and psychosocial factors (depression, education, socioeconomic residential area, and marital status). + Model 4 (random forest), accuracy (95%CI) of test data to predict CACS>0, including all CVD risk factors as in Model 2.

## Supplementary Materials

The following are available online at https://www.mdpi.com/1660-4601/17/3/778/s1, Figure S1: Social integration distribution of men in the pilot SCAPIS study. Shapiro–Wilkes *p*-value < 0.05. Figure S2: Social integration distribution of women in the pilot SCAPIS study. Shapiro–Wilkes *p*-value < 0.05. Figure S3: Emotional attachment distribution of men in pilot SCAPIS study. Shapiro–Wilkes *p*-value < 0.05. Figure S4: Emotional attachment distribution of women in pilot SCAPIS study. Shapiro–Wilkes *p*-value < 0.05. Figure S5: Bar plots of the prevalence of CACS = 0, CAC = 1–99, CAC score = 100–399, and CACS ≥400 according to different levels of social support in men. Data presented as %. *p*-values calculated by chi-squared tests for trend. Figure S6: Bar plots of the prevalence of CACS = 0, CACS = 1-99, CACS = 100-399 and CACS≥400 according to social support (participants with low social integration and emotional attachment) in women (A) and men (B). Data presented as %. P-values calculated by chi-squared tests for trend. Table S1: Questionnaires used to assess social support. Table S2: Prevalence of emotional attachment by social integration. *p*-values calculated using linear by linear association permutation tests. * *p* < 0.001. The number of discrepancy between the groups are explained by that participants completed questionnaires for social integration did not complete questionnaires for emotional attachment and vice versa. Table S3: Odds ratios, 95% confidence intervals, and p-values for CACS ≥ 100 and social support. OR, odds ratio; CI, confidence interval; OR, 95% CI, and p-values for CACS ≥ 100 and different levels of social integration and emotional attachment in the total study population and by sex after accounting for cardiovascular disease risk factors. ¤Model 1, all models adjusted for age and sex. ¤¤Model 2, adjusted for age, sex, and cardiovascular risk factor, including: family history of premature cardiovascular disease, burden of smoking (pack-years), systolic blood pressure, diastolic blood pressure, diabetes, obesity, HbA1C, low-density lipoprotein cholesterol, high-density lipoprotein cholesterol, and triglycerides.
