# Supplementary material for: Social Support and Subclinical Coronary Artery Disease in Middle-Aged Men and Women: Findings from the Pilot of Swedish CArdioPulmonary bioImage Study
Source: Int J Environ Res Public Health. 2020 Jan 27;17(3):778. doi: 10.3390/ijerph17030778 (PMC7037076; doi:10.3390/ijerph17030778)
Supplement: Supplementary file 1 [file ijerph-17-00778-s001.pdf]

International Journal of
Environmental Research and Public Health

Supplementary Materials: Social Support and Subclinical Coronary Artery Disease in Middle-Aged Men and Women: Findings from the Pilot of Swedish CArdioPulmonary bioImage Study

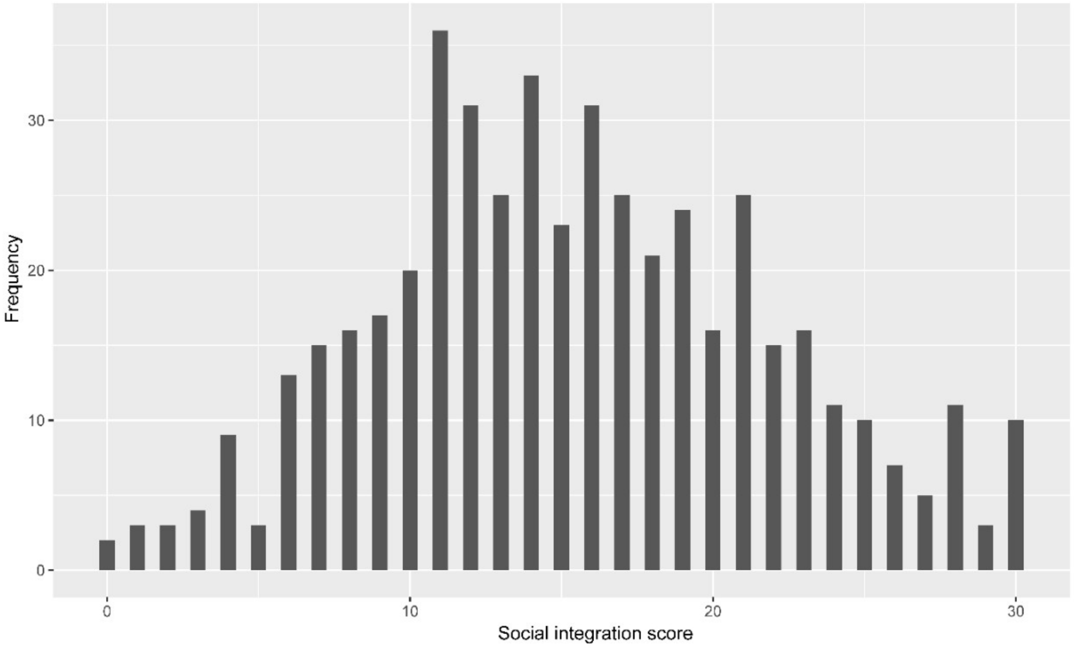

Figure S1. Social integration distribution of men in the pilot SCAPIS study. Shapiro-Wilkes *p*-value<0.05.

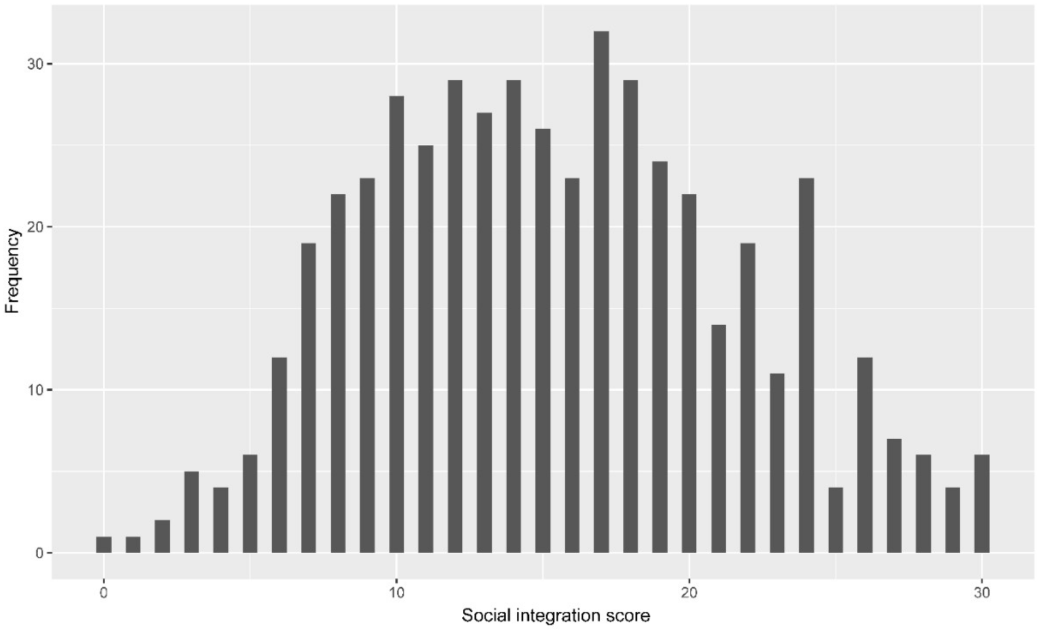

Figure S2. Social integration distribution of women in the pilot SCAPIS study. Shapiro-Wilkes *p*-value<0.05.

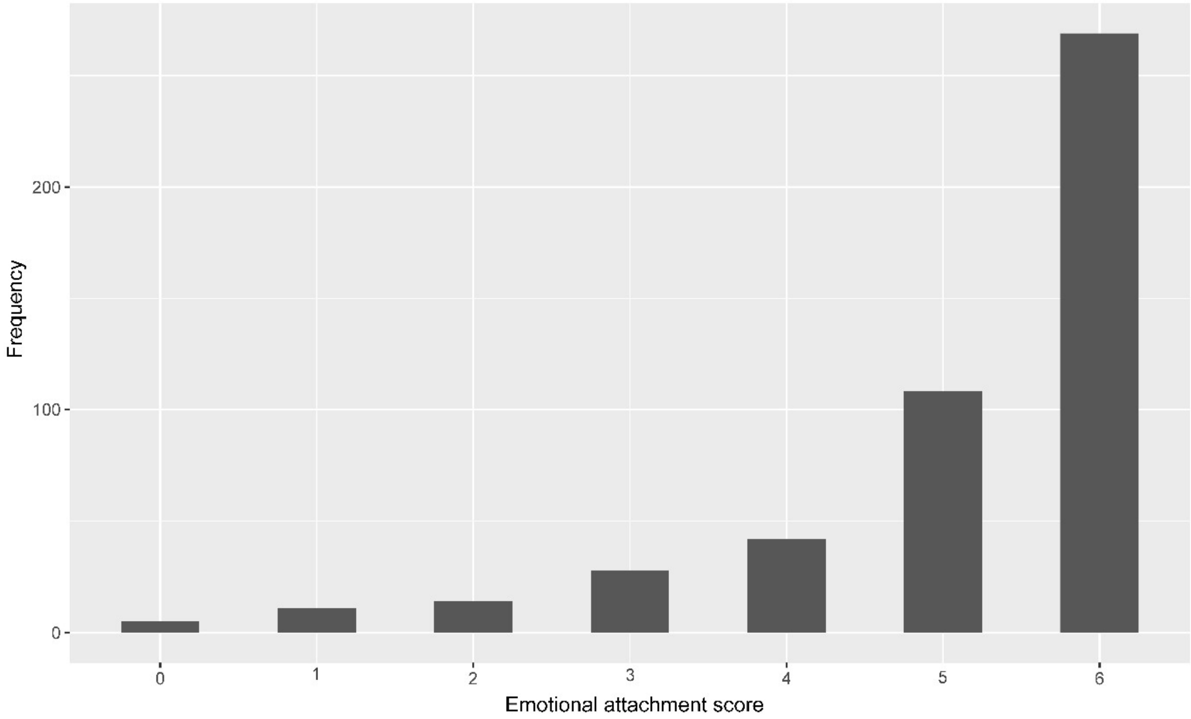

Figure S3. Emotional attachment distribution of men in pilot SCAPIS study. Shapiro-Wilkes *p*-value<0.05.

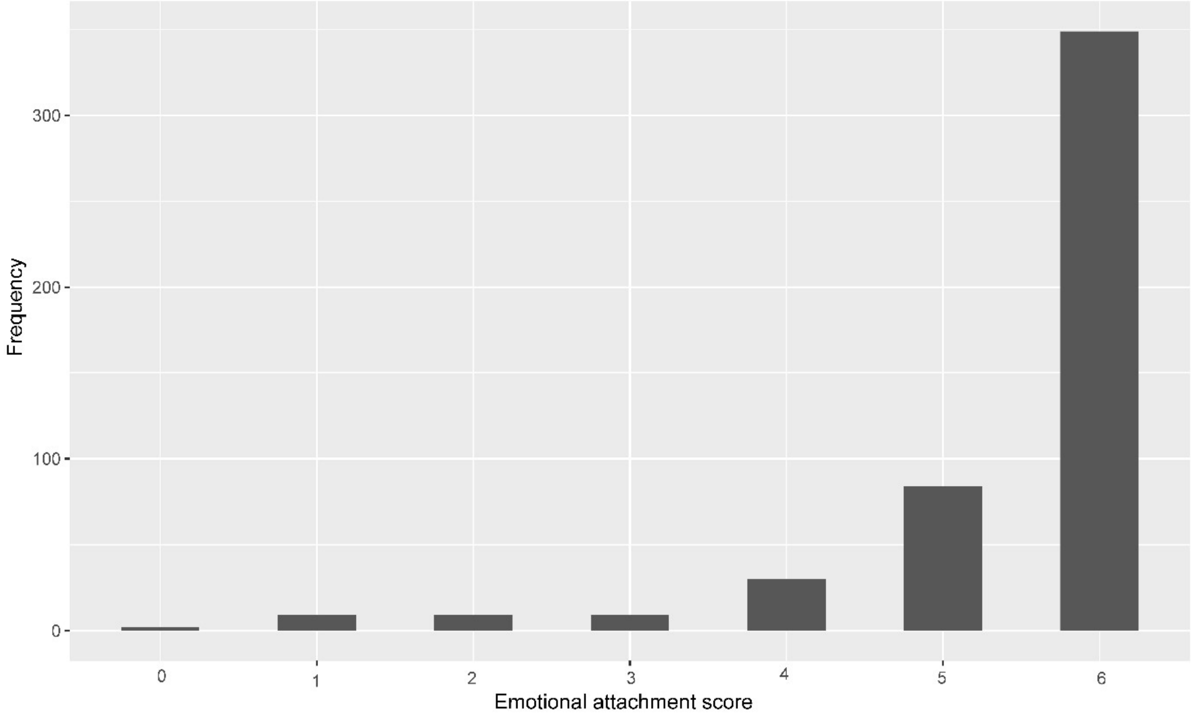

Figure S4. Emotional attachment distribution of women in pilot SCAPIS study. Shapiro-Wilkes *p*-value<0.05.

International Journal of
Environmental Research and Public Health

MDPI

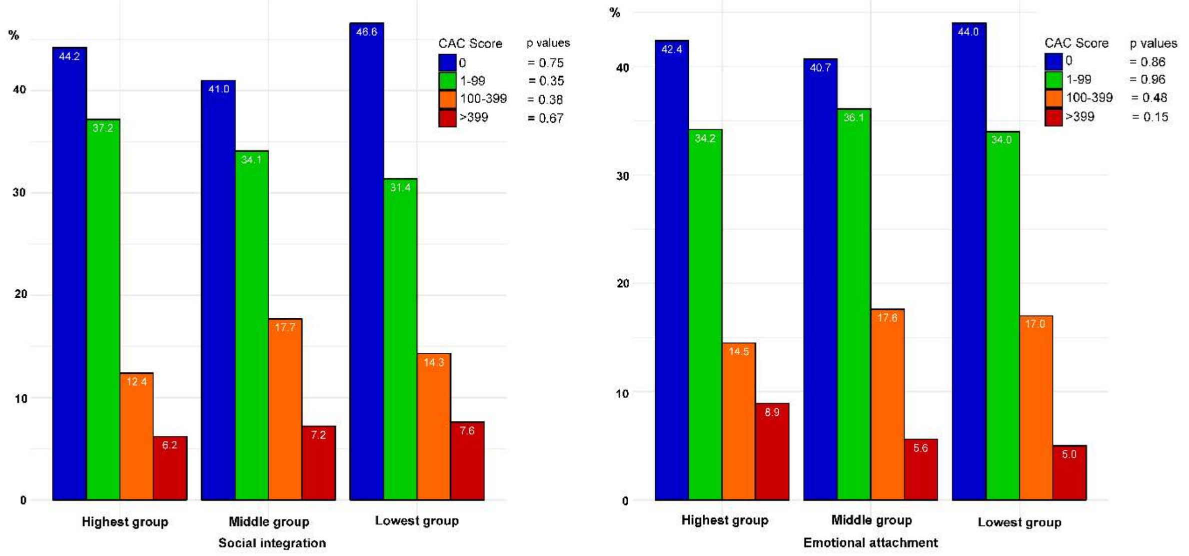

Figure S5. Bar plots of the prevalence of CAC score = 0, CAC = 1–99, CAC score = 100–399 and CACS ≥ 400 according to different levels of social support in men. Data presented as %. *p*-values calculated by chi-squared tests for trend.

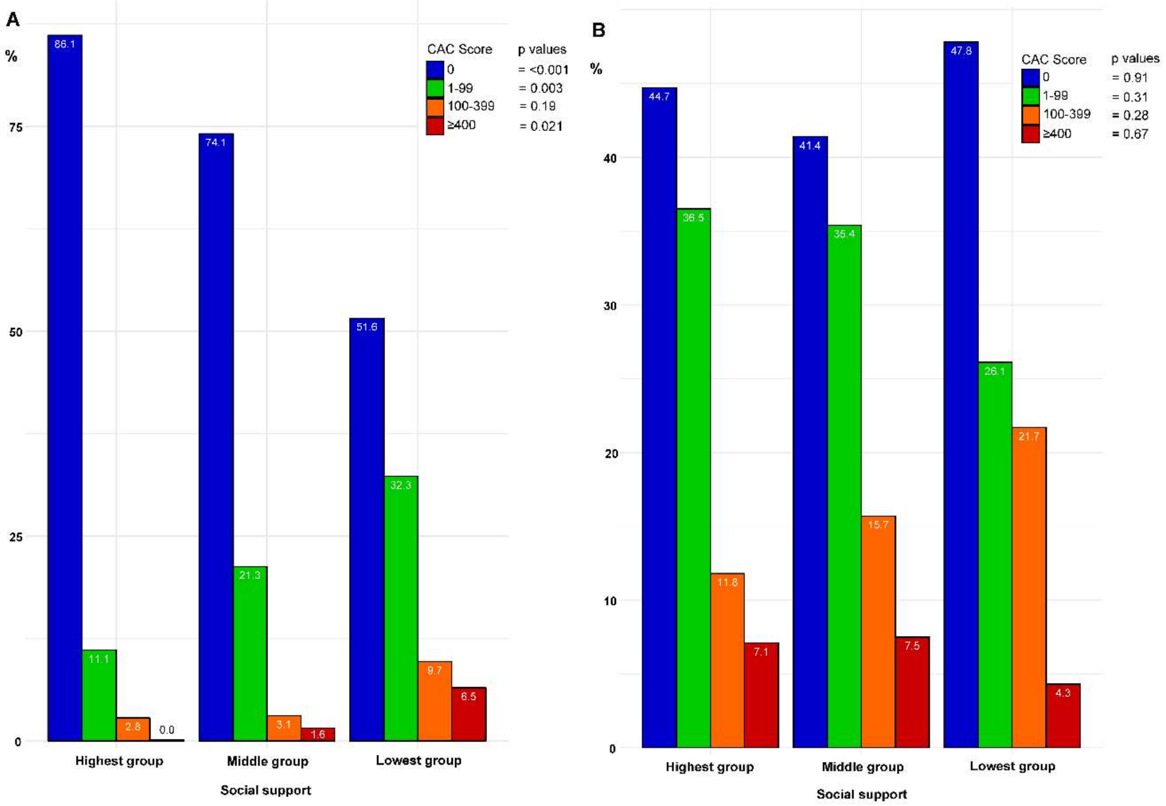

Figure S6. Bar plots of the prevalence of CACS = 0, CACS = 1–99, CACS = 100–399 and CACS ≥ 400 according to social support (participants with low social integration and emotional attachment) in women (**A**) and men (**B**). Data presented as %. *p*-values calculated by chi-squared tests for trend.

Table S1. Questionnaires used to assess social support.

**Social Integration**	**Optional Answers**
1) Number of people with whom respondent shares same interests	None, 1–2, 3–5, 6–10, 11–15, >15
2) Number of people met during a regular week	None, 1–2, 3–5, 6–10, 11–15, >15
3) Number of friends who at any time would come and visit respondent's home and who would not be embarrassed if it was untidy	None, 1–2, 3–5, 6–10, 11–15, >15
4) Number of friends or family members with whom respondent can talk openly	None, 1–2, 3–5, 6–10, 11–15, >15
5) Someone available whom the respondent can ask favors	None, 1–2, 3–5, 6–10, 11–15, >15
6) Someone available (apart from family) to whom respondent can turn in times of difficulties	None, 1–2, 3–5, 6–10, 11–15, >15
**Emotional attachment**	
1) Someone whom the respondent can lean on	Yes/no
2) Someone who feels very close to respondent	Yes/no
3) Someone to share feelings with respondent	Yes/no
4) Someone to confide in	Yes/no
5) Someone to hold and comfort the respondent	Yes/no
6) Someone at home, who appreciates what respondent does for him/her	Yes/no

Table S2. Prevalence of emotional attachment by social integration.

	**Men***			**Women***		
**Social integration**	**Highest (n = 129)**	**Middle (n = 249)**	**Lowest (n = 105)**	**Highest (n = 128)**	**Middle (n = 244)**	**Lowest (n = 123)**
Lowest emotional attachment group, n (%)	10 (7.8)	40 (16.0)	46 (43.8)	3 (2.3)	19 (7.8)	31(25.2)
Middle emotional attachment group, n (%)	20 (15.5)	69 (27.7)	15 (14.3)	15 (11.7)	38 (15.6)	29 (23.6)
Highest emotional attachment group, n (%)	85 (65.9)	128 (51.4)	37(35.3)	108 (84.4)	164 (67.2)	52 (42.3)

Notes. p-values calculated using linear by linear association permutation tests. * $p < 0.001$. The number of discrepancy between the groups are explained by that participants who completed questionnaires for social integration did not complete questionnaires for emotional attachment and vice versa.

International Journal of
*Environmental Research
and Public Health*

MDPI

Table S3. Odds ratios, 95 % confidence intervals and *p*-value for CACS≥100 and social support.

	Odds Ratio (95%CI)*	*p*-Value	Odds Ratio (95%CI)**	*p*-Value
**Social Integration**				
Highest group (total)	1.00		1.00	
Middle group (total)	1.44 (0.85–2.51)	0.19	1.25 (0.69–2.31)	0.47
Lowest group (total)	1.62 (0.86–3.07)	0.13	1.07 (0.52–2.20)	0.86
Highest group (women)	1.00		1.00	
Middle group (women)	1.84 (0.54–8.41)	0.37	1.16 (0.27–6.40)	0.85
Lowest group (women)	4.94 (1.43–22.90)	0.02	2.68 (0.56–15.85)	0.23
Highest group (men)	1.00		1.00	
Middle group (men)	1.38 (0.75–2.56)	0.31	1.27 (0.65–2.54)	0.49
Lowest group (men)	1.04 (0.50–2.17)	0.92	0.85 (0.36–1.99)	0.72
**Emotional attachment**				
Highest group (total)	1.00		1.00	
Middle group (total)	1.21 (0.70–2.06)	0.49	1.07 (0.58–1.94)	0.81
Lowest group (total)	1.19 (0.67–2.09)	0.53	0.89 (0.46–1.68)	0.72
Highest group (women)	1.00		1.00	
Middle group (women)	1.39 (0.43–3.89)	0.55	1.42 (0.36–4.97)	0.59
Lowest group (women)	3.53 (1.13–10.11)	0.02	1.43 (0.30–5.85)	0.63
Highest group (men)	1.00		1.00	
Middle group (men)	1.13 (0.60–2.09)	0.71	1.25 (0.63–2.47)	0.53
Lowest group (men)	0.88 (0.46–1.64)	0.69	0.70 (0.33–1.46)	0.35
**Social support**				
Highest group (total)	1.00		1.00	
Middle group (total)	1.41 (0.78–2.61)	0.27	0.98 (0.52–1.93)	0.96
Lowest group (total)	2.24 (0.95–5.25)	0.06	1.20 (4.52–3.09)	0.71
Highest group (women)	1.00		1.00	
Middle group (women)	1.79 (0.56–7.97)	0.37	1.15 (0.27–6.50)	0.86
Lowest group (women)	10.41 (2.16–59.21)	0.004	5.94 (0.71–52.73)	0.10
Highest group (men)	1.00		1.00	
Middle group (men)	1.38 (0.71–2.78)	0.36	1.17(0.57–2.46)	0.70
Lowest group (men)	1.33 (0.50–3.48)	0.56	0.93(0.30–2.77)	0.90

OR, odds ratio; CI, confidence interval; OR, 95% CI, and p-values for CACS >100 and different levels of social integration and emotional attachment in the total study population and by sex after accounting for cardiovascular disease risk factors. ¤Model 1, all models adjusted for age and sex. ¤¤Model 2, adjusted for age, sex and cardiovascular risk factor including: family history of premature cardiovascular disease, burden of smoking (pack-years), systolic blood pressure, diastolic blood pressure, diabetes, obesity, HbA1C, low-density lipoprotein cholesterol, high-density lipoprotein cholesterol, and triglycerides.
